# Dissociation of perception and motor execution of lower limb in multi-directional movements

**DOI:** 10.1038/s41598-023-44544-2

**Published:** 2023-10-11

**Authors:** Kyosuke Oku, Shinsuke Tanaka, Noriyuki Kida

**Affiliations:** 1 Graduate School of Human and Environmental Studies, Kyoto University, Kyoto, Japan; 2 Institute for Liberal Arts and Sciences, Kyoto University, Kyoto, Japan; 3 Faculty of Arts and Sciences, Kyoto Institute of Technology, Kyoto, Japan

**Keywords:** Human behaviour, Perception

## Abstract

Estimating the action capability is vital for humans to move their bodies successfully. Researchers have proposed reachability as an overestimation of motor abilities by judging unreachable distances as reachable. The existing literature has mainly investigated the sagittal direction, but multi-directional reachability is unexplored. This study examined the relationship between perception and motor using the reaching of the lower limbs in multiple directions. We asked 16 adults to reach targets projected onto the floor at 21 locations (seven directions and three distances) to estimate the reaching time. We found that the reaching time slowed as the direction increased toward the contralateral side, but the subjective reaching time did not change with direction. Multiple regression analysis showed that the subjective reaching time could be calculated accurately, mainly using the duration from the toe leaving the ground to movement completion. These results suggest that changes in direction may not be perceived precisely by the motor system of the lower limbs and that the subjective reaching time was strongly affected by the time after the toe left the ground. Our findings provide novel insights into the relationship between motor and perception in multiple directions, which may provide a new strategy for the maximal performance of lower-limb movement.

## Introduction

Daily, the central nervous system unconsciously adjusts the perception and motor skills to move the body effectively. To achieve precise motor adjustments, it is crucial to understand how our movements align with the surrounding environment. Thus, the central nervous system considers distance and direction to accurately calculate the necessary motor skills for effective and targeted body movements. Previous studies have investigated the ability to calculate the necessary perception and motor skills for performing targeted body movements by examining whether the anticipated movement was achieved. They have generally focused on upper-limb reaching movements and reported a tendency for individuals to judge unreachable distances as reachable^1,2^. These motor estimates vary according to one’s physical condition, whether the monocular or binocular vision is used^3^, and whether the upper body moves freely^4^. The surrounding environment also influences motor estimates, and estimation changes reportedly occur when an individual is rotated and subjected to a centrifugal force during horizontal reaching^5^. Scientific evidence thus shows that motor estimation is affected by physical conditions and the surrounding environment, indicating that accurate planning and effective body movement require an understanding of the relationship between perception and actual movement.

The relationship between the physical condition and the external environment is intricately connected to the direction of body movement. For instance, it is anticipated that motor estimations may differ depending on whether the movement is oriented toward the ipsilateral or the contralateral side. Studies have predominantly focused their analyses on three specific directions. However, there have been instances where academicians have included upward motion (reachable height), particularly emphasizing motions directed toward overhead targets^6^. This inclusion highlights the need for a comprehensive research approach that ensures direct comparisons across various directions. In several studies, the estimation was confined to a singular direction^3,7^. Given the diverse range of movements required in real-life scenarios, our research prioritizes the motor estimation of lower-limb movements across various directions, simulating conditions that mirror everyday activities.

Researchers studying motor estimates have examined both spatial and temporal factors. In studies investigating spatial factors, participants were asked to judge whether they could reach a target distance using the lengths of their arms^2,5^. Regarding temporal factors, a paradigm known as mental chronometry is used to verify the consistency between the actual response time and estimated response times. Research utilizing this paradigm has focused on walking time estimations^8^ and the time required for bimanual movements^9^. Researchers have demonstrated the effectiveness of mental chronometry in stroke rehabilitation^10^, and employed it as an evaluation metric for motor imagery before and after exercise training in post-stroke patients^11^. Furthermore, it has been suggested that a smaller difference between the estimated and actual response times indicates a higher motor imagery ability^12^. Motor imagery is “the mental expression of movement without actual bodily movement”^13^. In situations where one’s own movements must be judged within a limited timeframe, such as in sports, the temporal aspect is considered crucial. Therefore, we aimed to measure and estimate response time. Measuring the subjective response time and comparing it with the actual response time is expected to yield results more applicable to daily situations.

Studies on motor ability estimation have reported that various factors can lead to overestimating or underestimating motor capabilities. For example, there are differences in the estimation of walking time by age, with younger individuals tending to provide a relatively accurate estimation and older individuals overestimating (e.g., estimating a faster walking time)^8^. Moreover, younger individuals underestimate their ability to move certain distances^14^. They can reach overhead targets that they had believed were out of reach^6^. Other studies have reported accurate estimations of sports movements in basketball players, such as the height they can reach by jumping^15^. These findings suggest that various factors influence motor estimation, and consistent patterns of results for estimating one’s own movement have not yet been observed. Therefore, this study provides new insights into motor estimation by considering factors such as multi-directionality and multiple distances relevant to real-life and sports scenarios. Specifically, we focused on movements involving the lower limbs and investigated motor estimation using multi-directional and multi-distance approaches.

For multi-directional movements, the discrepancy between the sensory input and motor estimates increases as the center of pressure (CoP) shifts or changes its position relative to the lower limbs. For instance, when reaching the contralateral side of the lower limbs, the CoP shifts in that direction, which may influence movement accuracy and efficacy. Previous studies have shown that, during the first step of walking, the CoP transitions before the toes leave the ground^16–18^. Moreover, CoP transitions change with movement distance and direction^19^, albeit no report thus far has described the relationship between movements involving CoP transitions (e.g., whole-body movement) and motor estimation in multiple directions^20–22^. Based on these observations, we focused on the action capability in multiple directions using lower-limb movements involving CoP transitions.

**Figure 1:**
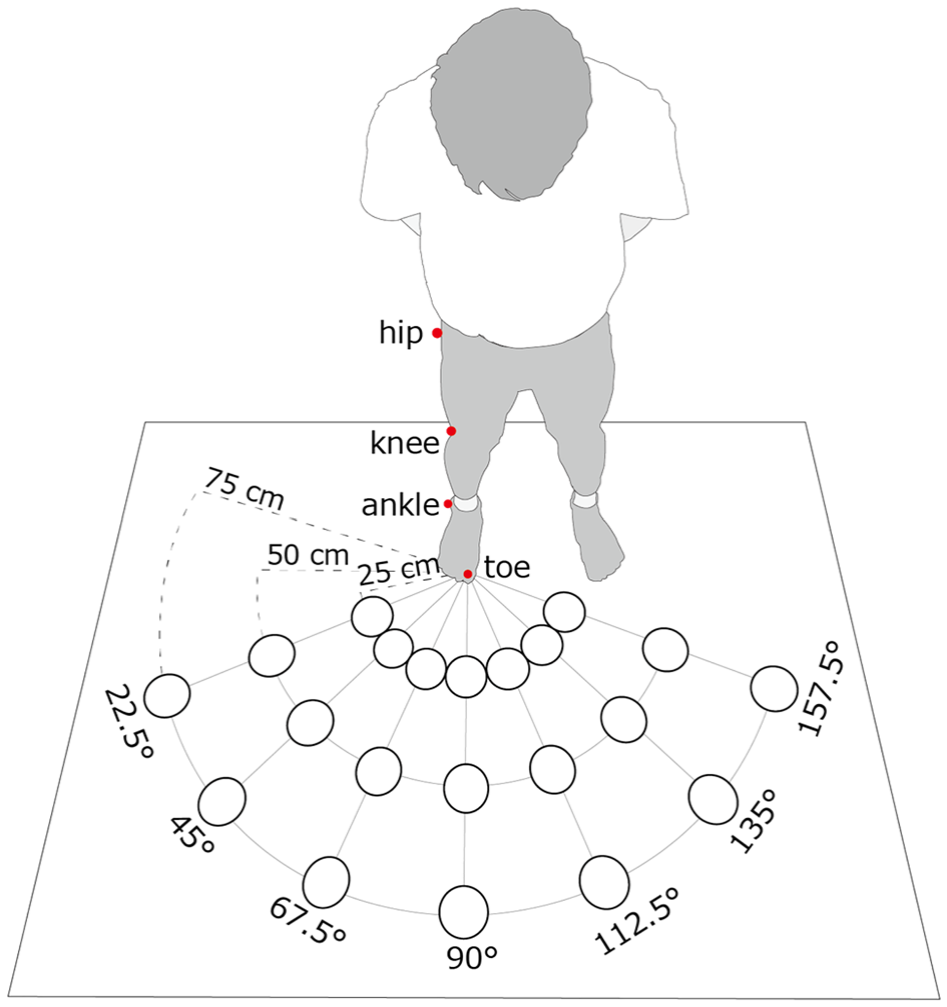
Overview of the experimental setup. The distance and direction of the projected visual stimulus and the location of the infrared marker. A visual target was presented in one of the 21 locations (seven directions and three distances) on the floor in front of the participant. It was used for both perception and motor tasks. An infrared marker was used to analyze actual movement. First, participants performed the motor task, reaching the target with their right foot. After completing the motor task, participants performed the perception task in which they judged without actual movement whether they could have reached the toe of the target before it went out. The motor and perception tasks comprised four blocks (each block containing 21 trials).

Specifically, we tested the relationship between movement time estimation and actual movement time in multiple directions through an experiment comprising perception and motor tasks. A schematic of the experiment is shown in Fig. 1. For the motor task, the participants had to reach the target with their right foot. A visual target was presented on the floor in front of the participant at one of 21 possible locations (seven directions and three distances). As soon as the participant visually perceived the target, the person attempted to reach the target while following the previously given instruction to “reach the toe on the target as quickly and accurately as possible.” To evaluate lower limb kinematics, we calculated the following indices: reaction time (RT), defined as the duration from target presentation onset to movement onset; movement time (MT), defined as the duration from onset to termination of the toe-reaching movement; and response time, defined as the sum of RT and MT, indicating the duration from target presentation onset to toe-reaching movement termination.

After completing the motor task, participants performed a perception task in which they judged, without actual movement, whether they would have been able to reach the toe on the target before the visual target went out. Similar to the motor task, the participant first stood upright on the platform and was then required to inform their own judgment for the same visual targets as in the motor task by pressing one of two buttons: “Yes” (judged to be reachable) and “No” (judged to be unreachable). The visual stimulus duration was shortened upon a “Yes” response and extended upon a “No” response. The procedure was repeated until a point of subjective equality (PSE) was reached, indicating the time the participant estimated that they could reach the toe on the target before the visual target went out with a 50% probability (see “Methods” section for further details).

Through these two tasks, we aimed to assess the relationship between the response time estimation and actual response time in various directions and distances. A smaller discrepancy between the estimated and actual response times suggests greater proficiency in motor imagery. Furthermore, we hope to provide fundamental knowledge of effective body movement control by investigating this relationship across different directions and distances. The findings of this study are expected to have practical applications in rehabilitation and sports coaching.

## Result

**Figure 2:**
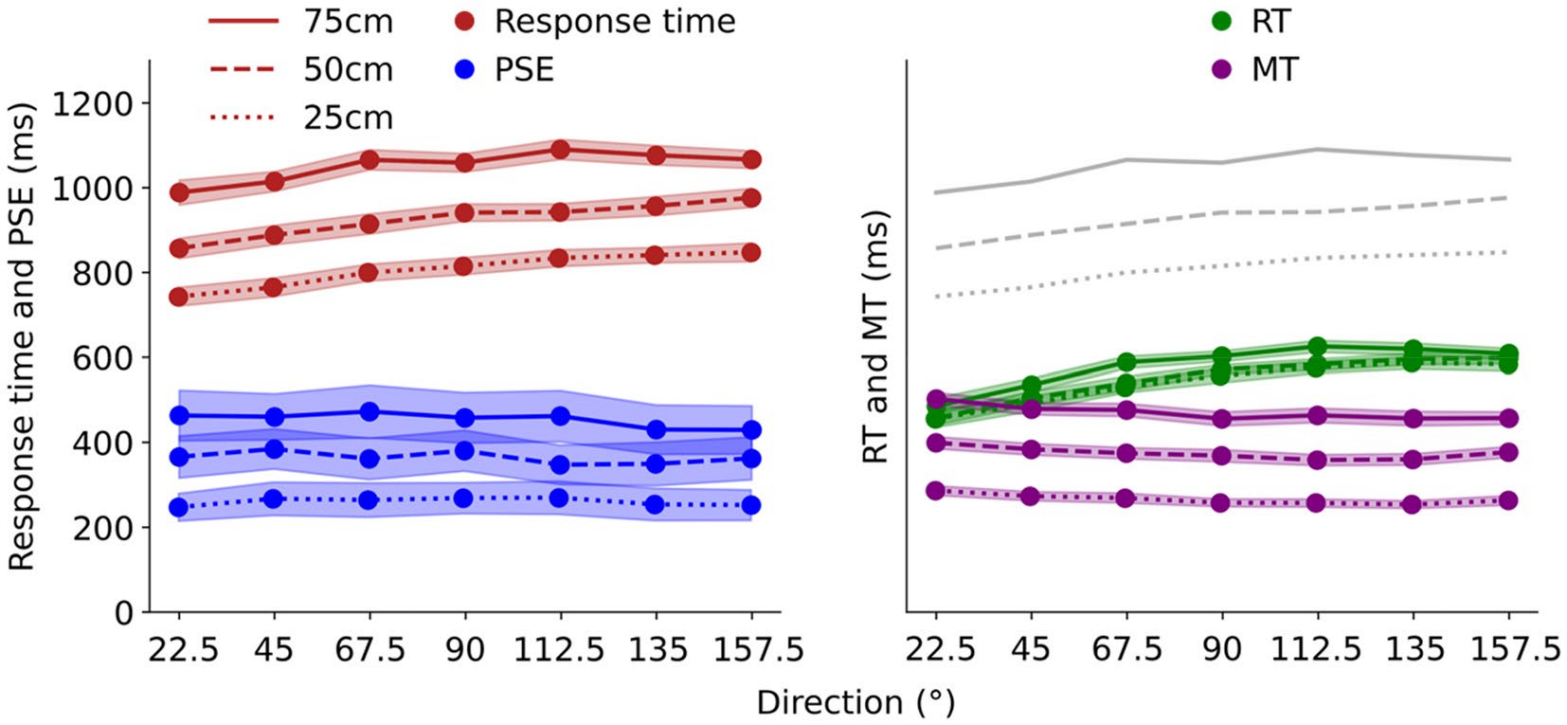
Left panel: Response time and point of subjective equality (PSE) to reach each direction. Right panel: The duration from visual stimulus presentation to the movement initiation of the toe in actual motion is referred to as reaction time (RT). The duration from the movement initiation to the completion is referred to as the movement time (MT). Response time is also shown in gray for reference. The lightly colored band indicates both panels’ standard error (SE). Response time, RT, and MT data were used from our previous study^23^.

We summarize the average PSE values in Table 1 and compare the response time from our previous study^23^ with the PSE values, as presented in Fig. 2. The differences between the response time and PSE and the mean values of the response time minus that of PSE for each direction and distance are summarized in Table 2.

**Table 1:** Mean values of the point of subjective equality (PSE) at each direction (°) and distance (cm). Standard errors (SE) are shown in parentheses.

Distance (cm)	Direction (°)						
	22.5	45	67.5	90	112.5	135	157.5
25	247.5 (129.9)	267.5 (157.3)	264.4 (162.1)	269.0 (146.7)	270.0 (157.0)	254.2 (152.6)	252.6 (144.7)
50	366.1 (198.5)	385.0 (189.3)	361.5 (195.2)	381.0 (192.5)	347.8 (185.5)	349.7 (205.8)	362.7 (201.8)
75	463.9 (238.3)	460.8 (217.4)	473.3 (248.8)	458.4 (238.6)	462.4 (241.7)	430.6 (233.1)	429.8 (227.4)

**Table 2:** Mean values of the difference between response time and PSE for each direction (°) and distance (cm). Standard errors (SE) are shown in parentheses.

Distance (cm)	Direction (°)						
	22.5	45	67.5	90	112.5	135	157.5
25	495.6 (41.0)	497.5 (54.6)	535.3 (49.4)	546.1 (45.2)	564.1 (49.9)	586.9 (46.3)	595.0 (47.2)
50	491.0 (58.6)	503.3 (58.5)	553.0 (56.5)	560.3 (52.8)	594.8 (49.3)	607.2 (59.7)	613.6 (58.5)
75	524.7 (67.6)	553.9 (55.5)	592.5 (61.0)	600.5 (59.3)	627.9 (58.8)	646.0 (61.5)	636.6 (61.3)

Table 2 shows that the difference between response time and PSE increases with the direction, especially toward the contralateral side. Subsequently, we conducted a three-factor analysis of variance (3 × 7 × 2 ANOVA), with distance (25 cm, 50 cm, and 75 cm), direction (22.5°, 45°, 67.5°, 90°, 112.5, 135°, and 157.5°), and task (motor and perception) as factors. The results did not reveal a significant two-way interaction effect (*F* (12, 180) = 0.33, *p* = 0.98, *η*^2^ = 0.00007), but a significant main effect of task (*F* (1, 15) = 116.84, *p* < 0.01, *η*^2^ = 0.72) was found. We conducted multiple Bonferroni-corrected comparisons and found a significant difference between response time and PSE at all 21 locations, indicating that the estimated values were smaller than the actual movement time.

To determine the relationship between directions, we conducted a correlation analysis for each kinematic parameter and perception, and the results are summarized in Table 3. The analysis indicated a significant positive correlation between the response time and direction for all distances. However, there was no correlation between the PSE and direction for any distance.

**Table 3:** Correlation between direction and each factor (kinematic parameter and perception).

	Distance (cm)		
	25	50	75
Response time	**0.40**	**0.39**	**0.28**
PSE	− 0.003	− 0.04	− 0.05
RT	**0.56**	**0.62**	**0.54**
MT	**− 0.21 (*p* < 0.05)**	− 0.17 (*p* = 0.07)	**− 0.22 (*p* < 0.05)**

Numbers in boldface denote statistical significance at *p* < 0.01.

The response time, point of subjective equality (PSE), reaction time (RT), and movement time (MT) were used in this study.

Response time, RT, and MT were obtained from our previous study^23^.

To further examine the body movements, we divided the response time into RT and MT; the average values are shown in Fig. 2, right panel. We conducted a correlation analysis between the kinematic factors (RT and MT) and the directions of the three distances (Table 2). The results showed a significant positive correlation between RT and direction for all distances. In contrast, a statistically significant negative correlation between MT and direction was observed at distances of 25 cm and 75 cm, with a tendency at 50 cm.

We conducted a multiple regression analysis to investigate the relationship between PSE and motor factors (RT and MT). We used the inter-participant means of RT and MT at each of the 21 locations as explanatory variables and the inter-participant means of PSE at each of the 21 locations as objective variables (N = 21). Consequently, we obtained Eq. (1), indicating a relationship between perception and movement with an adjusted R-square of 0.98, which demonstrated a highly accurate regression:

1 $$ {\text{PSE}} = 0.{91} \times {\text{MT}} + 0.{15} \times {\text{RT}}{-}{63}.{53} $$

RT and MT were significantly related, whereas the constant term was not significantly related (RT: coefficient = 0.91, *p* < 0.001; MT: coefficient = 0.15, *p* < 0.05; constant term: coefficient = − 63.53, *p* = 0.06).

## Discussion

Our findings suggest that perception involved in reaching movements of the lower limbs may not be accurately linked to actual motor skills. As illustrated in Fig. 2, the response time increases significantly in each direction. Nevertheless, the perception involved in the reaching movement (i.e., PSE) remained roughly constant, irrespective of the direction of movement. Thus, the difference between the PSE and response time increased in the direction toward the contralateral side throughout the reaching distance from 25 to 75 cm, as summarized in Table 2. In addition, ANOVA suggested an overestimation of action capability because PSE was always smaller than response time in all directions and distances tested, and the difference between perception and actual movement tended to become more prominent on the contralateral side. It can be speculated that the lower action capability toward the contralateral side could be largely attributed to a delay in RT because multiple regression analysis found a much lower contribution of RT as an explanatory variable compared to MT in estimating PSE (0.91 for MT versus 0.15 for RT). Therefore, the multiple regression analysis suggested that the motion of the MT could be perceived satisfactorily, whereas the movement of the RT specific to the lower limb may not be accurately perceived. The changes in the PSE and MT (Fig. 2) with increasing direction were similar, indicating that the subjective reaching time could be calculated and may be strongly influenced by the MT of the lower limbs.

The current study partially supports previous findings on the overestimation of action capability in the reaching movements of the upper limbs. Some studies have also reported that participants judged unreachable sagittal distances as reachable^1,3^. However, previous observations were based on overestimating the reachable target distance based on an individual’s body movements. Although there may be similarities between previous and current results regarding overestimation, this study further characterized the overestimation of motor ability using a time scale. Given that a real-life situation requires a limited execution time, the results of this study are more applicable to real-life settings.

This study examined lower limb movements in multiple directions, shedding light on variations in lower limb movement perception depending on direction and distance (Fig. 2). Accordingly, this study is the first to provide insights into the perception of the motor dynamics of the lower limb in multiple directions. Although some studies have been conducted on lower limb-related motor estimation^15^, the influence of movement direction is significant in multi-directional lower limb-related motor estimation—as suggested by the results of this study. Our findings reveal the possibility of some degree of bias in movement perception depending on the movement direction. For instance, the movement was not fully perceived across the entire spectrum of directions, leading to an overestimation of the action capability on the contralateral side.

As noted earlier, movement may not necessarily be reflected in perception, but it is reasonable to assume that the participants could imagine the movement. In this study, we employed a novel movement task that involved reaching the lower limbs. To control for prior knowledge of this type of movement, we recruited participants without experience in lower limb-specific sports, such as soccer. Subsequently, the participants performed a motor task before the perception task to prevent a situation in which they could not imagine or know too much about the movement of the lower limbs. Therefore, the perceptive distortions observed in this study can be attributed to the specificity of the lower limbs and not to the lack of imagery.

To better understand how response time changes with direction, we divided the response time into RT and MT and analyzed their correlation with direction. The results indicated that the RT increased (slowed down) with direction as the reaching task approached the contralateral side. In contrast, the MT decreased (sped up) with direction, as shown in Fig. 2. The separation of the response time into RT and MT provided a clearer understanding of their dependence in the opposite direction. Specifically, RT and MT showed highly positive and negative correlations, respectively, with direction. Furthermore, the correlation coefficient of RT against response time was much higher than that of MT, clearly demonstrating that variations in response time were more explicitly explained by changes in RT than by changes in MT, as illustrated in Fig. 2. In other words, the increase in RT with direction dominated the decrease in MT on the contralateral side, resulting in an overall increase in response time with direction, as depicted in gray in the right panel of Fig. 2.

Our findings regarding RT may be attributed to a variant of accelerated reaction time within the peri-personal space (PPS). The PPS is the space around the body^24^, and the reaction time for a stimulus is faster within the PPS^5,7,25^. The size of the PPS representation differs between body parts^26–28^. In this context, it has been confirmed that the PPS is also present in the lower limbs^29^, and the distance has been reported to be approximately 73 cm^30^. The farthest distance reached in this experiment was 75 cm, slightly greater than the reported value. Of the distances tested in this experiment, 50 cm and 25 cm were within the range of the reported PPS of the lower limb. Given the potential effect of PPS on RT, a track of 75 cm should deviate from that of 50 or 25 cm (Fig. 2). However, the changes in RT at 75 cm depicted the same curve along the direction as those at 50 and 25 cm (Fig. 2). This observation may contradict the view that PPS influenced the current results. Thus, it is unlikely that the RT will become faster only in certain parts owing to the effect of the PPS.

Furthermore, regarding the impact of attention on RT changes, it is believed that attention has a minimal influence on visual stimuli. Previous studies suggested that when performing bimanual movements, discriminative stimuli presented near the target location can be more accurately discriminated^31^. Additionally, attentional allocation allows for the effective discrimination of stimuli near multiple targets, even in continuous movement tasks^32^, and a similar allocation of perceptual resources around the target has been observed in grasping movements^33^. However, in this study, we ensured that perceptual resource allocation was not divided by pre-flashing the visual stimulus location three times before its onset and informing the participants. Thus, they initiate movements while fixating on the location of the visual stimulus during the movement initiation cue phase. Moreover, only a single visual stimulus was presented, making it unlikely that the perceptual resource allocation would be divided. Therefore, we believe that the influence of attentional allocation on the movements measured in this study was minimal.

### Limitations and future directions

This study investigated the estimation of reachability in a stationary environment; therefore, the results cannot be generalized to dynamic situations. For example, there may be a situation where judging whether a motor action is reachable or unreachable during its execution. In previous studies, time perception was distorted during motor execution^34,35^. This distortion also occurs when a visual target moves unpredictably^36,37^. As reachability must determine whether movement can be accomplished in a limited time, it may change under dynamic situations. Therefore, this study aimed to develop reachability in dynamic situations. Furthermore, in a previous study, the time series of interpersonal distance in Kendo was analyzed, and it was reported that the frequency of the relative phase varied with distance^38^. Similarly, the time series of interpersonal distance in soccer was also analyzed, and it was found that defenders won more often when the offense was more stable^39^. This study focused on the distances and time from a stationary state; however, in the future, we aim to investigate interpersonal distances and reachability in a dynamic situation. This will enable us to uncover more advanced human functions related to distance and time.

One limitation of this study was that it did not focus on spatial perspectives in measuring action capability. Instead, we primarily focused on the temporal aspect and assessed whether the movement was achievable. However, spatial action capabilities are crucial in everyday life and sports. Previous studies have often evaluated spatial reachability in movement estimation tasks^6,15^. Therefore, it may be beneficial to consider evaluating spatial aspects in future research, as this approach would enable a more comprehensive examination of reachability from various perspectives.

Another limitation of this study was the lack of electromyography (EMG) measurements. EMG examines the electrical activity of the muscles during movement initiation^40^. EMG has different concepts for the time from visual stimuli presentation to EMG activity onset (premotor reaction time) and the time from EMG activity onset to movement initiation (motor reaction time)^41^. Our findings showed a significant discrepancy between the actual movement time and the estimated time for contralateral direction movements. The main cause of this discrepancy may have been the increasing delay in the actual movement time as the movement progressed toward the contralateral direction. This study did not conduct EMG measurements; thus, we could not examine the premotor reaction time. Nonetheless, the premotor reaction time may influence the delay in reaching movements in the contralateral direction. Thus, researchers could conduct EMG measurements during lower limb reaching movements in multiple directions to investigate further the underlying causes of delayed movements in the contralateral direction. This approach can potentially allow for a more comprehensive examination of this phenomenon.

## Conclusion

The present study provides a novel insight that the reaching movement of the lower limbs may change with direction and cannot be perceived precisely, resulting in an overestimation of motor ability. For the actual movement, the reaching time became slower than the subjective reaching time on the contralateral side, and the differences between task completion and subjective reaching time increased as the direction increased. Kinematic data from our previous study^23^ demonstrated that movement preparation varies with direction, changing the timing of the toes leaving the ground. This characteristic was specific to the lower limbs, and the duration after the toe left the ground strongly influenced perception. Overall, our findings highlight the relationship between perception and movement, suggesting the involvement of joint transmission mechanisms in lower-limb movement, as discussed in our previous study^23^.

## Methods

### Participants

A total of 16 healthy adults (10 men and 6 women) aged 23.0 ± 2.55 years (mean ± SD) participated in this study. The participants were the same as those in a previous study^23^ and were asked to perform the task after completing the tasks in the previous study. All participants were right-footed, and footedness was established using the Waterloo Footedness Questionnaire^42^. Participants were selected based on the criteria that they had normal vision and no prior experience playing soccer at a competitive level. We selected the sample size (n = 16) based on previous studies that examined the relationship between motor function and perception^1,3,5,7^. Before the experiment, the purpose and procedure were explained to the participants, and written informed consent was obtained. This study was approved by the Ethics Committee of the Graduate School of Human and Environmental Studies, Kyoto University, and was conducted per the Declaration of Helsinki.

### Experimental setup and apparatus

A schematic of the experimental setup is shown in Fig. 1. The experimental setup used in this study is almost identical to that used in our previous study^23^. Performance was compared with that of the same group of actual motor movements. The participants stood upright on a wooden platform (180 cm wide × 90 cm deep) in a dim room. A projector (H6530BD, Acer) was placed approximately 200 cm above and 180 cm in front of the participant’s right foot and projected the visual targets onto the floor in front of the subjects. The visual target was a white circle 10 cm in diameter. It was drawn using custom software written in Visual Basic 2017 (Microsoft Visual Studio, Microsoft) running on a PC (Windows 10) using VGA outputs. A total of 21 visual targets in seven directions (22.5°, 45°, 67.5°, 90°, 112.5°, 135°, and 157.5°) and three distances (25 cm, 50 cm, and 75 cm) from the first toe of the right foot were used. The farthest distance from the visual target was set based on the approximate criterion that healthy adults can reach using a single reaching movement.

For the motor task, kinematic data were obtained using a motion capture system (OptiTrack and NeuralPoint). Reflective markers with a diameter of 4 mm were placed on the first toe of the right foot. The spatial locations were captured using six infrared cameras (OptiTrack Flex 3) with a temporal resolution of 100 Hz. An infrared LED (tip diameter of 5 mm and wavelength of 850 nm) placed on the floor was illuminated synchronously with the presentation of the visual target under the control of custom software to enable detection of the onset of visual stimulation by the motion capture system.

### Procedure

The experiment consisted of two tasks: perception and motor. First, participants performed a motor task to reach the target with their right foot. After the motor task, they performed a perception task in which they judged whether they could reach the toe on the target before it went out without making any actual movements. Both tasks consisted of four blocks (21 trials in each block), and short breaks (2 min) were inserted between the blocks to prevent fatigue. There was a practice session and a main session. The participants completed 10 motor task trials in the practice session before the main session. In contrast, they completed several trials in the practice session of the perception task until they could perform smoothly before the main session.

### Motor task

The participants stood upright with their heels on the platform and stabilized their posture before the task started. A visual target in one of the 21 locations (seven directions and three distances) was presented on the floor in front of the participant with a random delay of 1–4 s after the onset of the beeping cue at the target location. To minimize the visual search time, the presented target was pre-flashed three times to inform the participants of its location. As soon as the participant visually perceived the target, they attempted to reach it with their right foot, following the previous instruction to “reach the toe on the target as quickly and accurately as possible.” Upon reaching the target, the participants were asked to keep their toes on the target for 2 s to confirm the termination of the trial. The trials were consecutively repeated 21 times for all 21 locations, with a 5 s break between each trial in a block of the reaching task. A block of 21 trials was repeated four times, resulting in 84 trials.

### Perception task

Similar to the motor task, the participants stood upright on the platform and prepared to begin the task. A visual target was presented at one of the 21 locations (seven directions and three distances) on the floor in front of the participant. After the target presentation, the participants were required to judge whether they could reach the toe on the target before the projected target went out without making any reaching movements. Then they were required to inform their judgment by pressing either of the buttons that were “Yes” (judged to be reachable) and “No” (judged to be unreachable) on the handheld controller. Following the procedures of the parameter estimation by sequential testing (PEST), the presentation duration of the identical target for the next trial was shortened if the participant’s response was “Yes.” Simultaneously, it was extended if the response was “No”^43^. The procedure was repeated until a PSE was reached, indicating the time at which the participant was estimated to be able to reach the toe on the target before it went out, with a probability of 50%. For the PEST procedure, the initial duration of the target presentation was set to 400 ms with an initial step size of 200 ms. The step size halved every time the participant’s response was reversed from the previous one, such as from “Yes” to “No” or “No” to “Yes.” When the step size was greater than the duration of the target presentation, it was halved.

In some cases, the PSE measurement for the target was terminated when the step size reached 25 ms or less. The PSE for the target is represented by the average duration of the last two trials. If the participants answered “Yes” consecutively and the duration of the target presentation reached 50 ms, then the duration was returned to 100 ms, and the measurement was continued. Every response made by the participants was imported to the PC through a digital I/O board (PCI-2128, Interface) and stored as a text file.

### Data analysis

Time-series data of the 3-dimensional location of the reflective markers were preprocessed by applying a second-order Butterworth low-pass filter with a cutoff frequency of 10 Hz. The positional data were then differentiated over time using a three-point differential algorithm to obtain the velocity at each moment. The velocities in the three axes of each frame for each point were synthesized to induce instantaneous velocity in 3-dimensional space. The onset and offset of movements were defined using specific criteria. The first of five consecutive frames in which the velocity of the toe exceeded 30 cm/s was defined as the onset. In contrast, the last of five consecutive frames where the velocity fell below 30 cm/s was defined as the offset of movement. This threshold was set to 10% of the speed of the fastest movement among the 21 points, on average, for all participants.

To evaluate the kinematics of the lower limb, we calculated the following indices: RT, duration from target presentation onset to movement onset; MT, defined as the duration from onset to termination of the toe-reaching movement; and response time, defined as the sum of RT and MT, indicating the duration from target presentation onset to toe-reaching movement termination.

We conducted Shapiro–Wilk tests to assess the normality of the variables for the motor task (response time, RT, and MT) and perception task (PSE). We confirmed that they followed a normal distribution. Therefore, we analyzed each variable using the means and standard deviations. We further assessed the difference between the response time and PSE using a three-factor ANOVA, including three distances (25, 50, and 75 cm), seven directions (22.5°, 45°, 67.5°, 90°, 112.5°, 135°, and 157.5°), and two tasks (motor and perception). When a significant main effect was observed, the Bonferroni method was used for multiple comparisons. We also conducted a correlation analysis between each main variable (PSE, response time, RT, and MT) and direction to examine how movement and perception changed with direction. Multiple regression analysis was conducted with PSE as the dependent variable and each motor index as the independent variable to investigate the relationship between perception and motor tasks in greater detail. The prediction accuracy and coefficients of each independent variable were calculated.

## Data Availability

Data supporting the findings of this study are available upon request from the first author. The data are not publicly available because they contain information that could compromise the privacy of the research participants.
